# HBV Integration-mediated Cell Apoptosis in HepG2.2.15

**DOI:** 10.7150/jca.30493

**Published:** 2019-07-10

**Authors:** Xiaoge Hu, Jiahong Jiang, Chao Ni, Qiuran Xu, Song Ye, Junjie Wu, Feimin Ge, Yong Han, Yinyuan Mo, Dongsheng Huang, Liu Yang

**Affiliations:** 1 Key Laboratory of Tumor Molecular Diagnosis and Individualized Medicine of Zhejiang Province, Zhejiang Provincial People's Hospital, People's Hospital of Hangzhou Medical College, Hangzhou, Zhejiang 310014, P. R. China.; 2 Key Laboratory of Gastroenterology of Zhejiang Province, Zhejiang Provincial People's Hospital, People's Hospital of Hangzhou Medical College, Hangzhou, Zhejiang 310014, P. R. China.; 3 Department of General surgery, Zhejiang Provincial People's Hospital, People's Hospital of Hangzhou Medical College, Hangzhou, Zhejiang 310014, P. R. China.; 4 Division of Hepatobiliary and Pancreatic Surgery, Department of Surgery, The Secondary Affiliated Hospital, School of Medicine, Zhejiang University, Hangzhou, Zhejiang 310003, P. R. China.; 5 Department of Pharmacy, Zhejiang Provincial People's Hospital, People's Hospital of Hangzhou Medical College, Hangzhou, Zhejiang 310014, P. R. China.; 6 Department of Pharmacology/Toxicology and Cancer Institute, University of Mississippi Medical Center, 2500 North State Street, Jackson, MS 39216, USA.

**Keywords:** HepG2.2.15, viral integration, Hepatitis B virus, proliferation, cell cycle, apoptosis

## Abstract

Hepatocellular carcinoma (HCC) is the most common type of primary liver cancer and the second leading cause of cancer deaths in the word. Hepatitis B virus (HBV) infection plays an important role in the development of HCC. However, the mechanisms by which HBV integration affects host cells remain poorly understood. HepG2.2.15 cell line is derived from HCC cell line HepG2 with stable transfection HBV expression. In this study, HepG2.2.15 cells showed decreased proliferation, G1 cell cycle arrest and increased apoptosis, when compared to HepG2 cells. HBV capture sequencing was conducted in both genome and transcriptome level, followed by RNA expression sequencing in HepG2.2.15. Here, *CAMSAP2/CCDC12/DPP7/OR4F3* were found to be targets for HBV integration in both genome and transcriptome level, accompanied by alteration in their expression when compared to HepG2. Among these genes, *DPP7* was the only one gene with HBV integration into its exon, meanwhile *DPP7* expression level was also downregulated in HepG2.2.15 as compared to HepG2. Furthermore, *DPP7* knockdown resulted in increased apoptosis through upregulation of the Bax/Bcl2 ratio in HepG2 cells. Our results suggest that HBV integration of *DPP7* was involved in cell apoptosis.

## Introduction

The most prevalent primary liver cancer is known to be Hepatocellular carcinoma (HCC). It is considered to be the second highest cause of deaths associated with cancer globally 1, 2. Chronic infections with the hepatitis B virus (HBV) or hepatitis C virus (HCV) are the primary risk factors for HCC 3. In China, HBV plays a more prominent role because it may lead to liver fibrosis and cirrhosis, which eventually results in HCC 3-6. Around 50% of HCC patients are associated with HBV infections 7**.** Carriers of the hepatitis B surface antigen (HBsAg) are at 25-37-fold higher risk of developing HCC as compared to those not expressing the antigen 8, 9.

HBV contains eight known genotypes (A to H) 10. Its genome contains four genes, C, P, S and X, that encode the core protein (HBcAg), DNA polymerase, surface antigen (HBsAg) and protein X (HBx), respectively. Numerous studies have been done to establish the mechanisms of HBV-associated hepatocarcinogenesis 11-14. HBV genome integration, host gene deregulation and many viral factors have already been implied to contribute to HBV-associated HCC. HBx, for example, contributes to intracellular signal transduction, cell cycle and apoptosis regulation, and has been suggested to be an effector of hepatocarcinogenesis 11. Almost 90% of HBx transgenic mice develop HCC 15. HBV Integration leads to the dysfunction of host genes and affects cell proliferation, cell cycle progression, apoptosis, and even chromosomal stability 12, 16-20. Recurrent integrations in *TERT* and *MLL4*, for example, are frequently observed in HCC 21-29. HBx-LINE integration, which functions as a long non-coding RNA, has been shown to promote tumorigenesis of HCC 12. With the help of next generation sequencing, it has been found that genome-wide HBV integration is commonly reported in HCC 25, 30.

HepG2.2.15 cells are derived from HepG2 with HBV expression stablely^31^. Little work has been done in the context of HepG2.2.15 cells with regards to the effects of HBV on cellular function, and HBV integration detection of HepG2.2.15 cells has not been carried out. In this study, we conducted HBV capture sequencing in HepG2.2.15 cells at both the whole genome and transcriptome levels, followed by RNA sequencing. Our results reveal that several genes are targets for HBV integration, leading to altered expression patterns of these genes. Integration in the dipeptidyl peptidase 7 (*DPP7*) gene causes decreased expression of *DPP7* in HepG2.2.15 cells. Furthermore, *DPP7* knockdown results in increased apoptosis with upregulation of the Bax/Bcl2 ratio. Our results suggest that HBV integration of DPP7 was involved in cell apoptosis.

## Materials and Methods

### Cell culture and transfection

HepG2 was cultured with DMEM (HyClone, SH30022.01B) with 10 % FBS (Biological Industries) and incubated at 37℃ with 5% CO_2_. HepG2.2.15 was cultured with DMEM (HyClone) with 10 % FBS and 200ug/ml G418 (SIGMA). SiRNA transfection was done with Lipofection 3000 reagent according to the manufacturer's protocol. *DPP7* siRNA sequence 5'-3': siRNA#1 (CCGAGCACUGCUACGACAUTT), siRNA#2 (GCAACAAUGUGACCGAUAUTT), siRNA#3 (CCUGAGUGCCUCAGUCAUCTT).

### Antibody reagents

Protein expression was analyzed by western blot. Rb, pRb780, pRb795, pRb807/811, cyclinD, cyclinE, CDK2, p21, p27, p53, Bcl2, Bax antibody were obtained from Cell Signaling Technology (CST). *DPP7* antibody was bought from Santa Cruz Biotechnology. GAPDH antibody was bought from Beyotime.

### DNA and RNA isolation

With the use of Genomic DNA Mini Kit (Invitrogen, Life Technologies) and Trizol reagent (Takara), extraction of Genomic DNA and Total RNA from cells were carried out, respectively. Then, quantification of DNA and RNA was carried out with the use of NanoDrop 2000.

### HBV capture experiment at genomic DNA level

Firstly, HBV capture probe were designed based on full-length HBV genome of eight types (A to H). The sequencing library construction was conducted as per Illumina protocol. Genomic DNA was sheared to 150-200 bp DNA fragments by Covaris E-210. They were purified, end blunted, “A” tailed, and finally ligated to adaptors. 12 cycle PCR was carried out to amplify the ligation products to build the genomic DNA library. Library quantification was analyzed by Qubit dsDNA HS Assay Kit (Invitrogen). Next, libraries hybridization with HBV probe was carried out at 65℃ for 24 hours. Uncaptured fragments were then removed by washing., The eluted fragments underwent amplification by 16 cycles of PCR to generate sequencing library followed by paired-end index sequencing in the illumina HiSeq 2000 sequencer.

### HBV capture experiment at RNA level

Firstly, HBV capture probe were designed based on full-length HBV genome of eight types (A to H). As per Illumina paired-end sequencing protocols, 1 μg of total RNA was used to construct library. With ProtoScript® II Reverse Transcriptase (NEB), First-strand cDNA was synthesized. This was followed by second-strand cDNA synthesis with the help of Second Strand Synthesis Enzyme Mix (NEB) and purified using DNA Clean & Concentrator kit (Zymo Research). The double-strand cDNA products were sheared to 150-200 bp DNA fragments by Covaris E-210. These sheared fragments underwent purification, end blunting, “A” tailing, and finally ligation to adaptors. 12 cycles of PCR amplification were conducted for library construction. Libraries quantification was analyzed by Qubit dsDNA HS Assay Kit (Invitrogen). Then, library hybridization with HBV probe were conducted at 65℃ for 24 hours. Uncaptured fragments were removed by washing. Sequencing libraries were obtained by amplification of eluted fragments with 16 cycles of PCR, and were then subjected to paired-end index sequencing in the illumina HiSeq 2000 sequencer.

### Viral-Human Fusion identification by VirusSeq

VirusSeq 32 was used to detect viral-human fusion. Briefly, sequencing raw data was firstly filtered, so that clean reads could be obtained for subsequent analysis. The clean PE reads were then mapped onto both the human genome (NCBI build 37, HG19) and HBV genome (HE815465.1) by Burrows-Wheeler Aligner (BWA). hg19Virus is a novel hybrid reference genome made by combining human genome (H19) and virus genome (Virus). All PE reads are mapped to hg19Virus. Only the PE reads that mapped to both human chromosome and Virus, is considered as a discordant read pair. After annotation with human and viral genes, fusion candidates were reported for further analysis as previously reported 32. The coverage, depth and coverage rate onto HBV genome were measured according to reported reads.

### PCR and Sanger sequencing validation

For verification of the HBV integration breakpoints from VirusSeq analysis, Sanger sequencing was conducted. Using the paired-end assembled fragment as a basis, the PCR primers (Table S1) were designed, that one of the primers was located in HBV genome and the other in human genome. PCR was then carried out followed by sequencing.

### Detection of HBV gene copy number

HBV gene copy number was detected using Diagnostic Kit for Quantification of Hepatitis B Virus DNA based on manufacturer's protocol. Finally, PCR reactions of the prepared samples were performed with Stepone Real-Time PCR System.

### Cell proliferation analysis

We used Cell Counting Kit-8 (CCK-8, Biotool) for analysis of cell proliferation based on manufacturer's instruction. Cells were seeded in 96-well plates at a density of 3.5×10^3^ cells per well and cultured in DMEM with 10% serum. 24h/48h/72h later, the old medium was removed, and then 1/10 volume of CCK-8 was then added into 100ul fresh medium. After incubation at 37℃ for 4h, absorbance was measured at 450 nm.

### Cell cycle analysis

We used Cell Cycle and Apoptosis Analysis Kit (Beyotime) to analyze cell cycle based on manufacturer's protocol. Cells (1×10^6^) with different treatments were harvested followed by washing with cold 1 × PBS. Cells were then fixed in 1 ml 70 % cold ethanol at 4 ℃ for one night. Cells were washed three times with cold 1 × PBS, and then resuspended in 500 ul staining buffer (200 mg/ml RNase A and 50 μg/ml PI) and stained in the dark at 37 ℃ for 30min. Finally, Flow Cytometer (ACEA NovoCyte^TM^) was used for cell cycle detection.

### Cell apoptosis analysis

We used Annexin V-FITC/PI apoptosis kit (Multi Science) to analyze cell apoptosis based on manufacturer^'^s protocol. Cells (5 × 10^5^) with different treatments were harvested followed by washing with cold 1 × PBS. The cell were resuspended in 500ul 1 × Binding Buffer, and 5 μl Annexin V-FITC and 10 μl PI were then added to the cell mixture followed by incubation in the dark at 37 ℃ for 5 minutes. Finally, apoptosis was detected by Flow Cytometer (ACEA NovoCyte^TM^).

### RNA extraction, reverse transcription and qPCR

We used Trizol (Takara) to extract RNA from cells. cDNA synthesis was conducted by PrimeScript^TM^ RT Master Mix kit (Takara). PCR reaction was then done with SYBR Premix Ex Taq^TM^, (Tli RNaseH Plus) kit (Takara). The primers used in this paper were shown in Table S2. GAPDH was used as a control gene.

### Statistics

Data were presented as mean ± SEM based on three independent experiments. Unpaired student's t test analysis was performed in quantification of RT-PCR results. *p* values that were less than 0.05 were considered to be statistically significant.

## Results

### Detection of HBV in HepG2.2.15 cells

**Figure 1:**
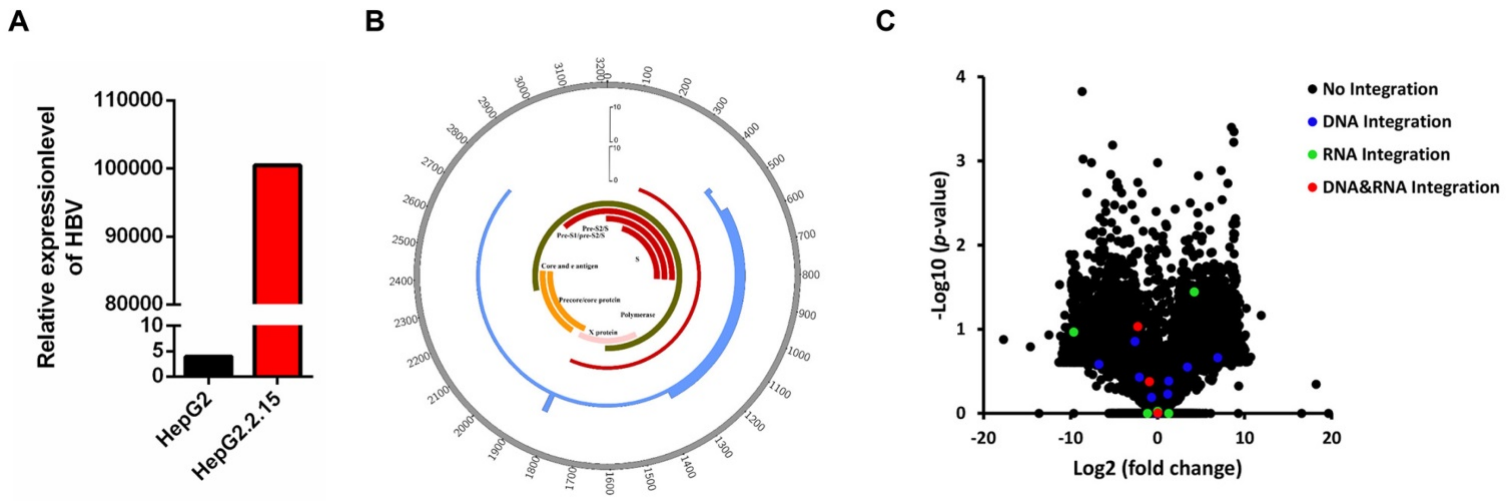
** HBV integration breakpoints distribution in HBV genome and gene expression change in HepG2.2.15.** (A) Detection of HBV gene copy number by PCR. (B) Distribution of integration breakpoints in the HBV genome in HepG2.2.15 (genome integration breakpoints of HepG2.2.15: blue, transcriptome integration breakpoints of HepG2.2.15: red) are shown. Histograms were constructed for 100bp intervals. HBV genes with different functions are shown. (C) Volcano plot of differently expressed genes between HepG2.2.15 and HepG2. The logarithms of the fold changes of genes (x-axis) are plotted against the negative logarithm of their p-value(y-axis). Black dots represent genes without HBV integration (No integration), blue dots represent genes with HBV integration in genome (DNA integration), green dots represent genes with HBV integration in transcriptome (RNA integration), red dots represent genes with HBV integration in both genome and transcriptome (DNA&RNA integration).

HBV DNA was detected in HepG2.2.15 cells by a fluorescent labeled PCR probe. HepG2 cell was served as a negative control. As shown in Figure 1A, the HBV DNA copy number in HepG2.2.15 cells showed a 25,763-fold increase in comparison with HepG2, confirming that HepG2.2.15 cells are positive for HBV DNA.

### Identification of genome HBV integration sites

**Figure 2:**
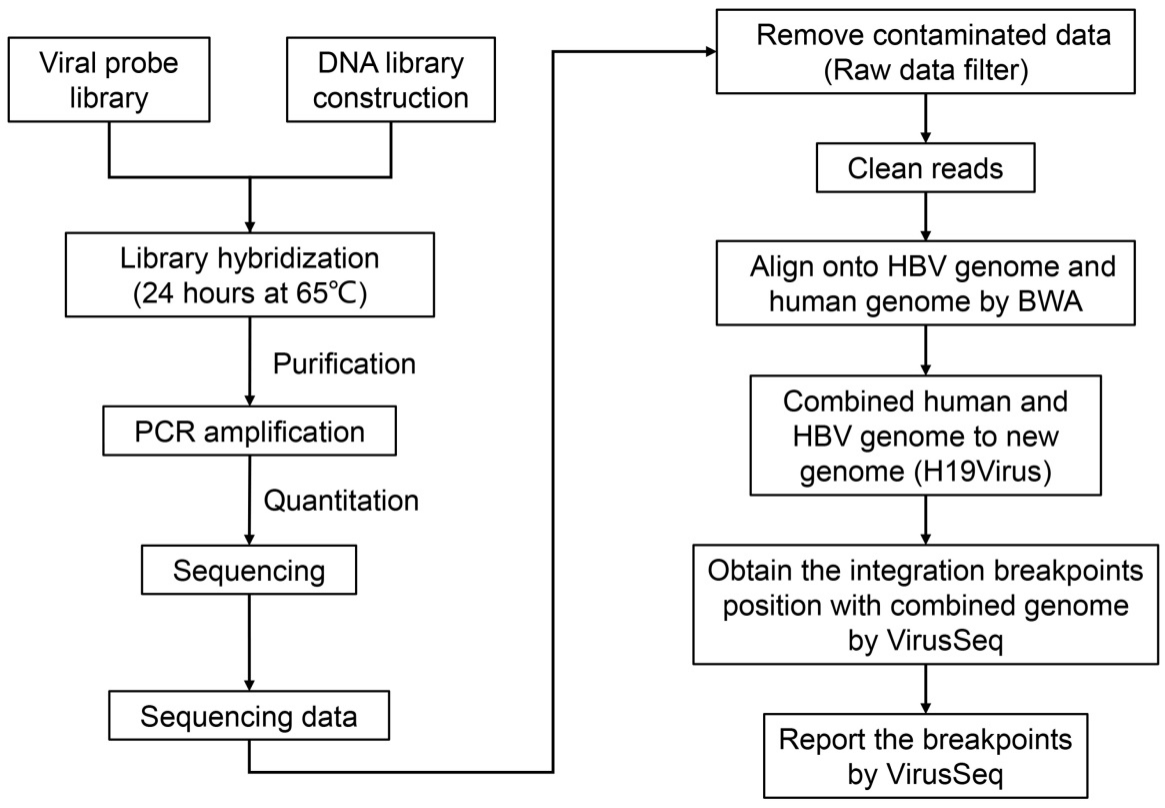
** The pipeline of workflow.** Experimental workflow and bioinformatic analysis is performed in this study. BWA: Burrows-Wheeler Aligner.

To explore the integration of the HBV genome in HCC, we conducted HBV capture sequencing in HepG2.2.15 cells using the Illumina HiSeq 2000 sequencing system. Identification of the integrated HBV genome was subsequently performed using VirusSeq 32 (Figure 2).

We obtained 2,374,482 raw reads that had with a 141 bp average read length from HepG2.2.15 genomic DNA samples, of which 192,901 reads were matched to the HBV genome (HBV-D-3) (Table 1). An average depth of 6417.52 and 100% coverage was achieved with 20-fold read mapped to the HBV genome (Table 1). 14 HBV integration breakpoints were identified (Table 1), and the genome HBV integration breakpoints in HepG2.2.15 were distributed in HBV genome (Figure 1B).

As shown in Table 2, 14 HBV integration breakpoints are mainly located within the region where the X protein, S protein and core protein are located 33. The HBx protein is the most frequently integrated protein. HBV is preferentially integrated into chromosome 1 (4/14), chromosome 2 (3/14), chromosome 3 (2/14), chromosome 7 (1/14), chromosome 9 (1/14), chromosome 20 (1/14), chromosome 21 (1/14) and chromosome X (1/14) (Table 2). The majority of HBV integrations are intronic and intergenic, while the *DPP7* integration sites are exonic (Table 2).

### Identification of transcriptome HBV integration sites

To further explore HBV integration at the level of the transcriptome, total RNA of HepG2.2.15 cells was obtained, reversed transcribed to cDNA and subjected to HBV capture sequencing (Figure 2).

We obtained 94,192,016 raw reads with an average read length of 138 base pairs. A total of 561,510 reads were matched to the HBV genome (HBV-D-3) (Table 1). We obtained 100% coverage of the targeted region with 20-fold read mapped to the HBV genome (Table 1). Nine HBV integration breakpoints were identified (Table 1), and the transcriptome HBV integration breakpoints in HepG2.2.15 were distributed in HBV genome (Figure 1B).

As shown in Table 3, all nine HBV integration breakpoints are distributed in the region where the X protein and S protein are located within the HBV genome 33 (Table 3). Three integration breakpoints are within the S protein and six are within the X protein. The HBV integration sites are located within chr1, chr3, chr5, chr7, chr9, chr11, chr12, chr17 (Table 3). Most of the integrations are intronic and intergenic, while the HBV integration of *DPP7* is exonic (Table 3).

### Expression of target genes with HBV integration

To explore whether HBV integration affects the expression of host genes, we conducted RNA-Seq in HepG2 and HepG2.2.15 cells. Statistical analysis of RNA-Seq data is shown in Table 4. Expression change of genes were shown in Figure 1C, including the genes without or with HBV integration in genome level, transcriptome level and both genome and transcriptome level. Combining the RNA-Seq and HBV integration results, four genes (*CAMSAP2/CCDC12/DPP7/OR4F3*) are identified with HBV integration in both the genome and transcriptome of HepG2.2.15 cells (Table 5). Expression of *DPP7*, which shows integration of the X protein transcript, decreases 4.8-fold as compared to expression in HepG2 cells. Changes in expression levels of *CAMSAP2/CCDC12/OR4F3*, which show intronic or intergenic HBV integration, are lower (Table 5). To confirm HBV integration into the genes, PCR amplification followed by Sanger sequencing was carried out. Integration of HBV into the four genes (*CAMSAP2/CCDC12/DPP7/OR4F3*) was confirmed by Sanger sequencing (Figure S1), validating the results from HBV capture sequencing.

### HBV integration decreases cell proliferation, and induces G1 arrest and apoptosis

**Figure 3:**
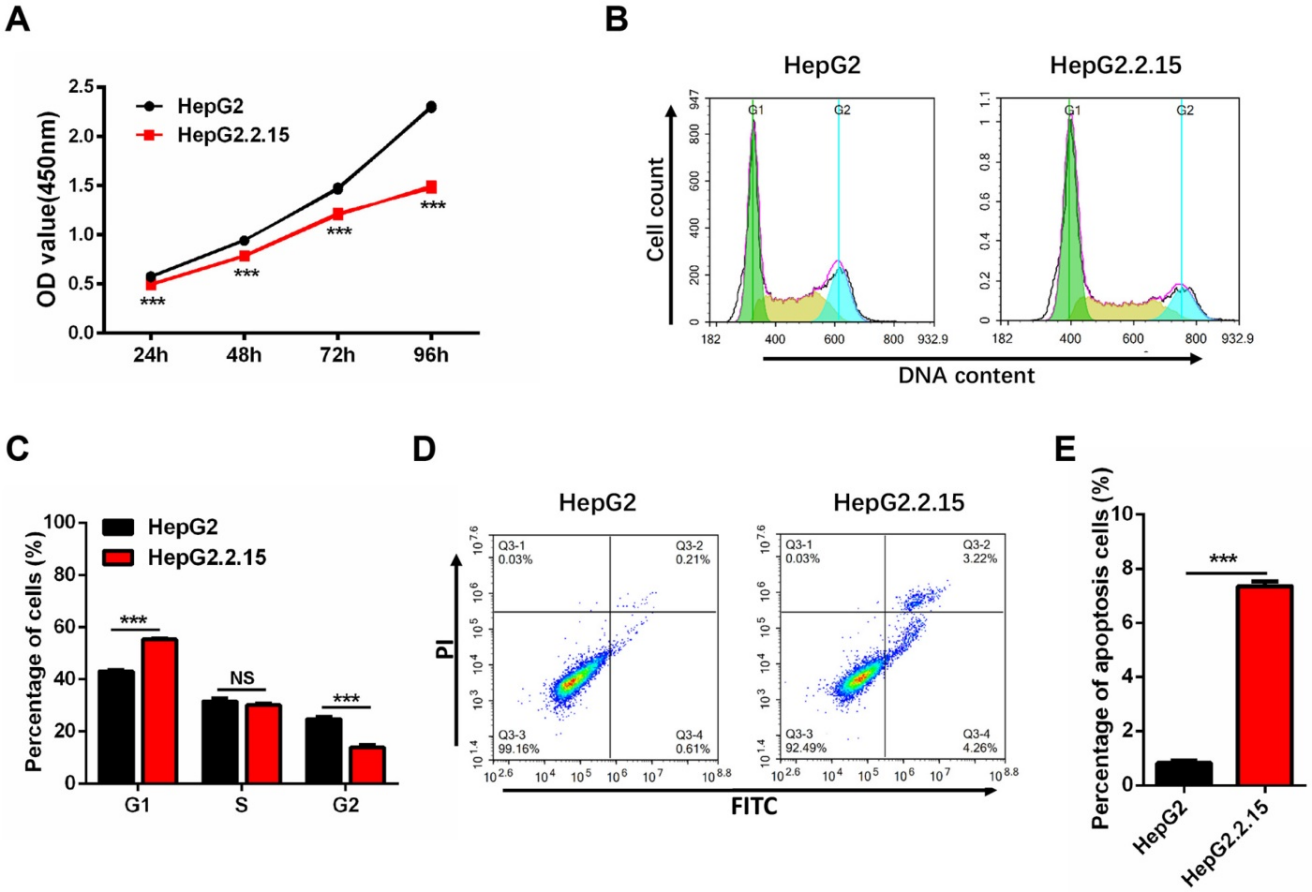
** HepG2.2.15 cells show decreased proliferation, G1 cell cycle arrest and increased apoptosis.** (A) Proliferation of HepG2 and HepG2.2.15 cells. OD450 values were measured at 24 h, 48 h, 72 h and 96h. Data shown represents the mean ± SEM values from three independent experiments (***p < 0.001). (B) Cell cycle analysis of HepG2 and HepG2.2.15 cells. (C) Quantitative cell cycle distribution of HepG2 and HepG2.2.15 cells. HepG2.2.15 cells show G1 cell cycle arrest when compared to HepG2 cells. Data shown represents the mean ± SEM values from three independent experiments (***p < 0.001, NS: none significant). (D) Analysis of cell apoptosis by flow cytometry. Increased apoptosis is observed in HepG2.2.15 cells. (E) Quantitative results of cells undergoing apoptosis in HepG2 and HepG2.2.15 populations. Data shown represents the mean ± SEM values from three independent experiments (***p < 0.001).

Since HepG2.2.15 cells originate from HepG2 with HBV expression stablely, we first examined whether the cell phenotype is stable. The cell proliferation assay shows a significant reduction of HepG2.2.15 cells growth rate as compared to HepG2 cells (Figure 3A). Cell cycle analysis was conducted to determine whether cell cycle distribution was changed. As compared to HepG2 cells, there was an obvious increase in the proportion of G1 phase and a decrease in the S and G2 phases in HepG2.2.15 cells (Figure 3B, C). The decreased cell proliferation and G1 cell cycle arrest are consistent with previous reports 34. Cell apoptosis detection was also carried out by flow cytometry, and the rate of apoptosis in HepG2.2.15 cells is increased in comparison with HepG2 (Figure 3D, E).

**Figure 4:**
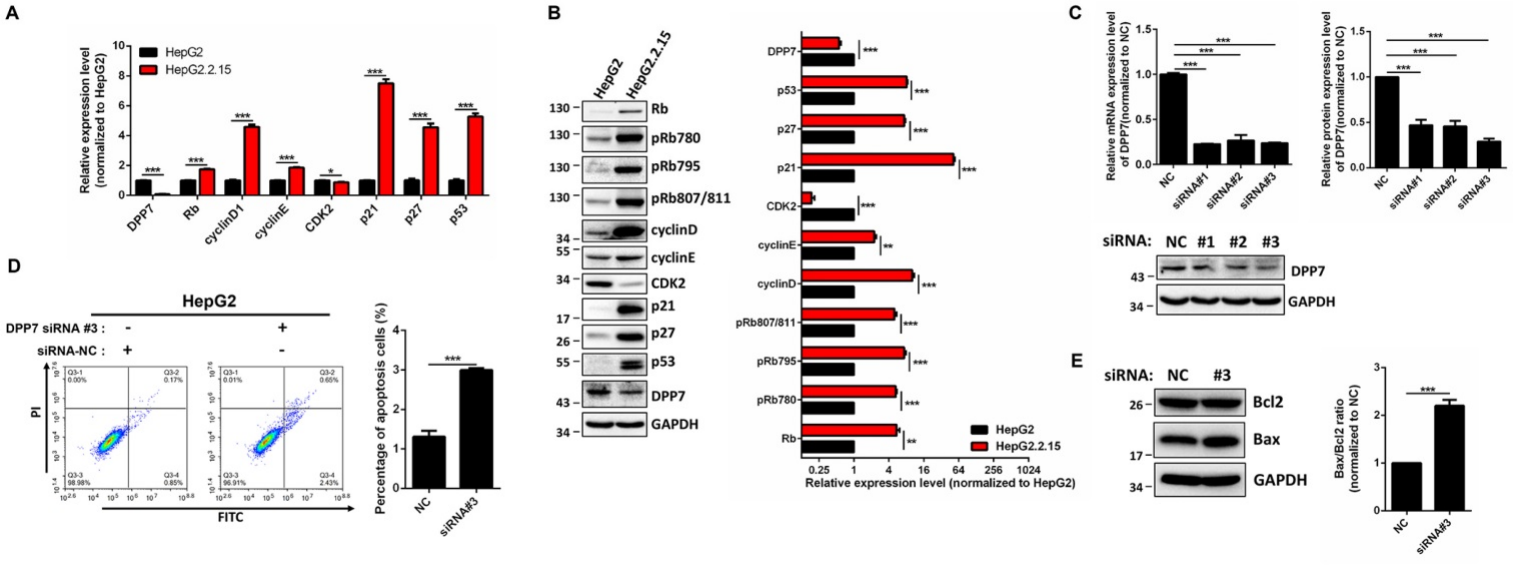
** DPP7 knockdown induces cell apoptosis in HepG2 cells** (A) RT-PCR analysis of cell cycle regulated genes in HepG2 and HepG2.2.15 cells. GAPDH was used as a reference gene. Data shown represents the mean ± SEM values from three independent experiments (*p < 0.05, ***p < 0.001). (B) Western blot analysis of cell cycle regulated genes. Quantification results were shown in the right. GAPDH was used as a reference gene. Data shown represents the mean ± SEM values from three independent experiments (**p<0.01, ***p < 0.001). (C) Verification of *DPP7* siRNA knockdown efficiency by RT-PCR and western blot. Quantification results were shown in the upper. Data shown represents the mean ± SEM values from three independent experiments (***p < 0.001). (D) Knockdown of *DPP7* induces increased cell apoptosis in HepG2 cells. Data shown represents the mean ± SEM values from three independent experiments (***p < 0.001). (E) Knockdown of *DPP7* in HepG2 cells leads to increased Bax/Bcl2 ratio as measured by western blot. Quantification results were shown in the right. GAPDH served as a loading control. Data shown represents the mean ± SEM values from three independent experiments (***p < 0.001).

To identify the mechanism of G1 cell cycle arrest, expression of cell cycle-regulated genes were detected. The results obtained from RT-PCR showed that mRNA expression of Rb, cyclin D1, cyclin E1, p21, p27, and p53 is increased in HepG2.2.15 cells, while expression of *CDK2* is decreased (Figure 4A) in comparison with HepG2 cells. In addition, the protein expression of Rb, cyclin D1, cyclin E1, p21, p27, p53 is upregulated, while there was a downregulation of *CDK2* expression in HepG2.2.15 cells (Figure 4B).

### *DPP7* knockdown results in cell apoptosis

In order to determine whether the phenotype of HepG2.2.15 cells is linked with HBV integration, we studied *DPP7*, since it is the only gene with HBV integration at both the genome and transcriptome levels, and decreased expression levels. The mRNA expression of *DPP7* is 10-fold decrease in HepG2.2.15 in comparison with HepG2 (Figure 4A).

*DPP7*, also referred to as quiescent cell proline dipeptidase (QPP, DPP2, DPPII), is a post-proline cleaving aminopeptidase 35, 36. It has been previously reported that *DPP7* inhibition in quiescent lymphocytes results in cell apoptosis 37. We reasoned that the HBV integration-mediated decreases in *DPP7* expression may be associated with cell apoptosis in HepG2.2.15 cells. Therefore, we used *DPP7* siRNAs for knockdown of *DPP7* (Figure 4C). In HepG2 cells, *DPP7* knockdown induced more cell apoptosis than the siRNA control (Figure 4D). Apoptosis-related proteins such as Bcl2 and Bax play an vital role in cell apoptosis. We then analyzed the expression of apoptosis-related proteins to explore the cellular mechanism of the higher apoptosis of HepG2.2.15 cells. Western blot results show that *DPP7* knockdown results in an upregulation of Bax, without affecting Bcl2 (Figure 4E). This leads to an increase in Bax/Bcl2 ratio, which may contribute to the increased levels of apoptosis.

## Discussion

Currently, HCC is the second leading cause of cancer-associated deaths globally. In China, HBV has a vital contribution to HCC development. The HepG2.2.15 originates from HepG2 by stable transfection with HBV genome 31. In comparison with HepG2, HepG2.2.15 shows decreased proliferation, G1 cell cycle arrest and increased rate of apoptosis. In our study, HBV capture sequencing was conducted at both the genome and transcriptome levels to discover HBV integration sites in the HepG2.2.15 genome. RNA-Seq was subsequently performed to analyze gene expression. Based on the HBV capture sequencing and RNA-Seq results, a number of genes show HBV integration accompanied by altered expression. In particular, integration of HBV into *DPP7* is observed both at the genome and transcriptome levels, and expression of this gene is reduced in HepG2.2.15 cells when compared to HepG2 cells. Furthermore, *DPP7* knockdown leads to apoptosis with upregulation of the Bax/Bcl2 ratio in HepG2 cells, which suggest that HBV integration-induced decreases in *DPP7* expression can increase apoptosis in HepG2.2.15 cells, indicating that HBV can regulate cell function through integrating into host genes.

Previous studies have revealed a large number of genes with HBV integration in human HCC tissue 30. Although a large amount of data has been obtained in studies of clinical samples, few studies have performed functional analysis. The mechanism by which HBV integration induces HCC remains to be elucidated. One of the challenges in identifying these mechanisms is that HBV was randomly integrated into the genome, which makes choosing a cell line model with specific HBV integration difficult. HBx-LINE1, an HBV-human chimeria contribute to HCC through acting as a LncRNA 12. In the aforementioned study, six HCC cell lines were obtained from patients who were infected with HBV, which paves the way for further functional studies 12. Here, we chose HepG2.2.15 cells as our HBV integration cell model, to explore the relationship between HBV integration and cell function and phenotype.

In a previous study, whole-genome gene expression were profiled in both HepG2 and HepG2.2.15 cells. It showed that 2978 genes are up- or down-regulated, including genes related to cell cycle, transport, signal transduction, cell adhesion and cellular metabolism 38. Two-dimensional gel electrophoresis-mass spectrometry indicated that HBV infection can affect protein expression 39, 40. HepG2.2.15 cells also show reduction in proliferation and G1 cell cycle arrest with alterations in cell cycle-regulated genes 34, 41, 42. However, the association between HBV integration and cell functional and phenotypic changes has not been elucidated. In our study, HepG2.2.15 cells showed reduction in proliferation, G1 cell cycle arrest and increased apoptosis when compared to HepG2 cells. HBV capture sequencing and RNA-Seq revealed that HBV integration occurs in *DPP7* accompanied by decreased expression levels of the gene. *DPP7*, a post-proline cleaving aminopeptidase, has been shown to play role in apoptosis of lymphocytes 37. Consistent with this finding, we show that *DPP7* knockdown leads to increased apoptosis with upregulation of the Bax/Bcl2 ratio in HepG2 cells. Together, our data suggests that HBV integration affects the expression of host genes such as *DPP7*, leading to increased apoptosis in HepG2.2.15 cells.

Although our results reveal alterations of several important genes, it has some limitations. For example, many genes have been identified with HBV integration in HCC tissue, whereas fewer genes were detected here, which may be attributed to the cell model we choose. Since HepG2.2.15 cells originate from HepG2 with HBV expression stablely, they do not exactly mimic the HBV infection process under physiological conditions. In this regard, HepG2.2.15 may not be the best cell line model for HBV integration analysis. Nevertheless, this cell line provides a tool to study HBV integrations and the effects on expression of host genes. Our results suggest that HBV infection decreases host cell activities, such as decreased cell proliferation, and increased cell apoptosis, which are contrary to the predominant hypothesis that HBV infection contributes to hepatocarcinogenesis 4-6. HBV contains eight genotypes (A-H) with different geographic distribution. Increasing evidence has suggested that not all HBV genotypes are linked with HCC 43, 44. The HBV genotype of HepG2.2.15 cells is genotype D, 31 and the clinical impact of genotype D is still controversial. For example, some clinical and epidemiological data suggests that genotype D does not affect the severity and chronicity of the disease 45, while other studies report that genotype D is related to chronic and occult HBV infections 46. In these previous studies, a large amount of data was obtained from clinical specimens and, therefore, more functional studies are needed to clarify these issues. It is possible that the decreased cell proliferation and cell activity induced by HBV integration in HepG2.2.15 is specific for HBV genotype D, as previously reported 34.

## Supplementary Material

Supplementary figure and tables.

## Figures and Tables

**Table 1:** HBV capture sequencing data

Sample	Reads number	Raw bases (Mb)	Clean bases (Mb)	Average read length	No. of the reads mapped to HBV	Target 20X rate (%)	Target mean depth	Coverage rate (%)	No. of integration breakpoints
Genomic DNA	2374482	358.55	316.91	141	192901	100	8157.27	100	14
Total RNA	94192016	14222.99	12390.66	138	561510	100	22604.45	100	9

**Table 2:** HBV genomic integration breakpoints and gene expression fold change

Human location	Nearest genes	Location type	HBV location	HBV transcript	Supporting reads number	Nearest gene fold change (log2)
chr1:155104681	EFNA1	intronic	460	S protein	14	-0.693
chr1:200809469	CAMSAP2	intronic	1746	X protein	8	-0.923
chr3:47015291	CCDC12	intronic	1820	X protein	71	0.023
chr9:140008668	DPP7	extronic	1716	X protein	114	-2.261
chr2:27241835	MAPRE3	intronic	1829	X protein	11	-2.092
chr1:200809321	CAMSAP2	intronic	1746	precore/core protein	10	-0.923
chr1:569999	OR4F3	intergenic	1801	precore/core protein	13	-
chr7:25573057	NPVF	intergenic	1594	X protein	103	0
chr3:42696821	ZBTB47	intronic	1829	X protein	112	-2.59583
chrX:9148656	FAM9B	intergenic	2082	precore/core protein	55	-6.74895
chr20:62935214	LINC00266-1	intergenic	2639	polymerase protein	38	3.4362
chr2:27775260	GCKR	intergenic	2775	polymerase protein	22	6.90382
chr21:10007085	TEKT4P2	intergenic	607	S protein	45	1.28415
chr2:27915039	SLC4A1AP	intronic	2567	polymerase protein	22	1.17871

Chr: chromosome; -: no signal test.

**Table 3:** HBV RNA integration breakpoints and gene expression fold change

Human location	Nearest gene	Location type	HBV location	HBV transcript	Supporting reads number	Nearest gene log2(fold change)
chr11:85195011	DLG2	intronic	407834	S protein	9	1.283
chr5:71146794	MAP1B	intergenic	408128	S protein	8	4.212
chr17:33478111	UNC45B	intronic	407658	S protein	6	-1.162
chr1:200809476	CAMSAP2	intronic	409112	X protein	12	-0.923
chr3:47015261	CCDC12	intronic	409144	X protein	41	0.023
chr9:140008690	DPP7	exonic	409079	X protein	359	-2.261
chr7:25572926	MIR148A	intergenic	408983	X protein	16	0.003
chr1:570051	OR4F3	intergenic	409270	X protein	8	-
chr12:20704358	PDE3A	intronic	408967	X protein	7	-9.627

Chr: chromosome; -: no signal test.

**Table 4:** Statistics information of RNA-seq data

Sample	Reads number	Raw bases (Mb)	Clean bases (Mb)	Average read length	Average insert size	Mapped reads	Coverage rate(%)	Target mean depth
HepG2	193531404	29223.24	25439.31	130	948.7	96922102	62.76	59.55
HepG2.2.15	145773306	22011.77	19359.6	126	989.9	98707630	61.12	65.79

**Table 5:** HBV integration genes in both genome and transcriptome

Gene	Chr	HBV transcript	Integration type	fold change (log2)
CAMSAP2	chr1	X protein/core protein	intronic	-0.923
CCDC12	chr3	X protein	intronic	0.023
DPP7	chr9	X protein	exonic	-2.261
OR4F3	chr1	X protein/core protein	intergenic	-

Chr: chromosome; -: no signal test.
