# Supplementary material for: HBV Integration-mediated Cell Apoptosis in HepG2.2.15
Source: J Cancer. 2019 Jul 10;10(17):4142–50. doi: 10.7150/jca.30493 (PMC6692610; doi:10.7150/jca.30493)
Supplement: Supplementary file 1 — Supplementary figure and tables. [file jcav10p4142s1.pdf]

Supplementary Files:

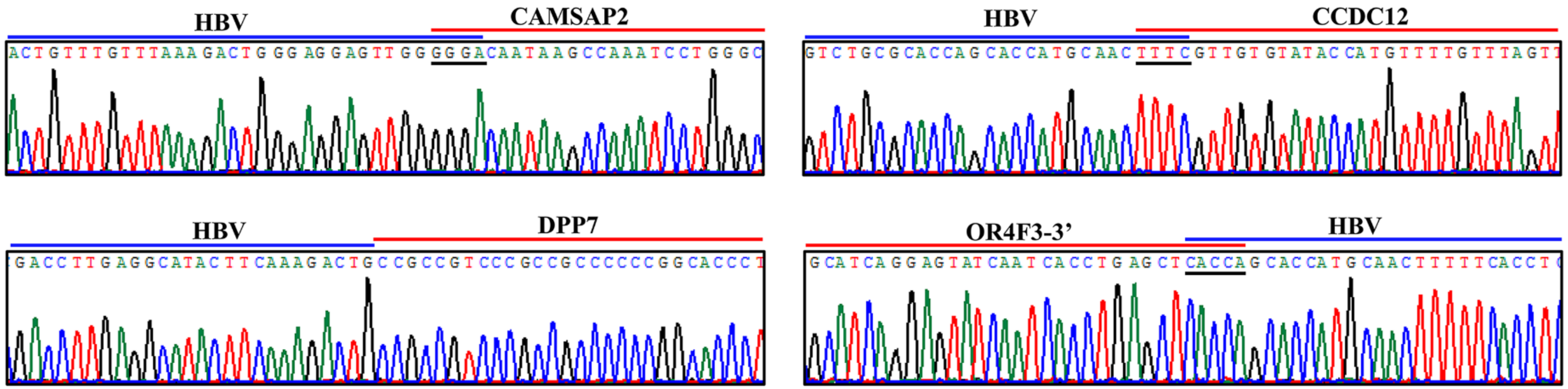

Figure S1. Sequence verification of HBV-human integration breakpoints by Sanger Sequencing. HBV-human fusions in both the genome and transcriptome were validated by Sanger sequencing of samples generated from RNA. Alignment of the human and HBV sequences around the integration sites are shown.

Table S1. Sequences of primers used for Sanger sequencing.

Gene	Primer orientation	sequence （5’ - 3’）
CAMSAP2	Forward	CTTCGCTTCACCTCTGCACG
	Reverse	GTTTGAGGAGGCTGAGGAGGA
CCDC12	Forward	CGTGAACGCCCACCGAA
	Reverse	CGCACCAGGCCTTGTACTC
DPP7	Forward	CGGGGCGCACCTCTCTT
	Reverse	TGCTCCGCGAAGACCAGTAGA
OR4F3	Forward	TACCACTTCAGCCTAGCCCCTAC
	Reverse	AGCTCCAAATTCTTTATAAGGGTC

Table S2. Sequences of primers used for quantitative RT-PCR analysis.

gene	Primer orientation	sequence （5’ to 3’）
DPP7	Forward	GGTGTCGGACAGGTTCTGG
	Reverse	GCGAAGACCAGTAGAGCCC
Rb	Forward	ACTCTCACCTCCCATGTTGC
	Reverse	TGCACTCCTGTTCTGACCTC
cyclinD1	Forward	CCCTCGGTGTCCTACTTCA
	Reverse	CTCCTCGCACTTCTGTTCCT
cyclinE	Forward	CAGCCTTGGGACAATAATGC
	Reverse	TTGCACGTTGAGTTTGGGTA
CDK2	Forward	CAGGATGTGACCAAGCCAGT
	Reverse	TGAGTCCAAATAGCCCAAGG
p21	Forward	TTAGCAGCGGAACAAGGAGT
	Reverse	CGTTAGTGCCAGGAAAGACA
p27	Forward	ATGTCAAACGTGCGAGTGTCT
	Reverse	CCGAGCTGTTTACGTTTGACG
p53	Forward	TAGTGTGGTGGTGCCCTATG
	Reverse	CCAGTGTGATGATGGTGAGG
GAPDH	Forward	GATTTGGTCGTATTGGGCG
	Reverse	TGGAAGATGGTGATGGGAT
